# Angiotensin Converting Enzyme Inhibition and Mortality in Pulmonary Hypertension Associated With Chronic Obstructive Pulmonary Disease (PH‐COPD)

**DOI:** 10.1002/pul2.70348

**Published:** 2026-07-16

**Authors:** Athiththan Yogeswaran, Meike Fünderich, Katharina Wendlandt, Jeffrey S. Annis, Evan Brittain, Christina A. Eichstaedt, Ekkehard Grünig, Hector R. Cajigas, Robert Frantz, Andrew J. Sweatt, Roham T. Zamanian, Friedrich Grimminger, Jochen Wilhelm, Hossein‐Ardeschir Ghofrani, Khodr Tello, Werner Seeger, PVRI GoDeep Consortium, Mauro Acquaro, Imad Al Ghouleh, James Anderson, Anastasia Anthi, Alexandra Arvanitaki, Aparna Balasubramanian, Felix Ballmann, Joan Albert Barbera, Harm Jan Bogaard, Hugh Buzacott, John Cannon, Demircan Ceren, Stephen Y. Chan, Alejandro Cruz Utrilla, Victoria Damonte, Eckart De Bie, Juliana De Luque Figueroa, Effrosyni Dima, Philipp Douschan, Nathan Dwyer, Diego Echazarreta, Jean Elwing, Pilar Escribano Subias, Kai Förster, Marlize Frauendorf, Stefano Ghio, George Giannakoulas, Lars Harbaum, Paul M. Hassoun, Melanie Heberling, Anne Hilgendorff, Luke Howard, Patrick Janetzko, Arun Jose, Ernesto Junaeda, Cihangir Kaymaz, David G. Kiely, Ingrid King, Hans Klose, Ziad Konswa, Grzegorz Kopec, Gabor Kovacs, Philipp Krieb, Keiichiro Kuronuma, Edmund Lau, Melanie Lavender, Allan Lawrie, Mona Lichtblau, Raphael W. Majeed, Kurt Marquardt, Hiromi Matsubara, Farhan Mubashir, Horst Olschewski, Mauricio Orozco‐Levi, Karen Osborn, Antonella Pepe, Joanna Pepke‐Zaba, Alba Ramirez‐Sarmiento, Timo Rosenbach, Stephan Rosenkranz, Hani Sabbour, Sandeep Sahay, Khaled Saleh, Laura Scelsi, Yuriy Sirenko, Siva Sivakumaran, Thenappan Thenappan, Ioan Tilea, Olena Torbas, Mark Toshner, Silvia Ulrich, Andrea Varga, Helen M. Whitford, Christoph B. Wiedenroth, Martin R. Wilkins, Paul G. Williams, Shaun Yo, Nayeli Zayas, Zhenguo Zhai, Zhu Zhang

**Affiliations:** ^1^ Department of Internal Medicine Universities of Giessen and Marburg Lung Center (UGMLC), Member of the German Center for Lung Research (DZL) Giessen Germany; ^2^ Institute for Lung Health (ILH) Cardio‐Pulmonary Institute (CPI) Giessen Germany; ^3^ Department of Infectious Diseases, Respiratory Medicine and Critical Care Charité‐Universitätsmedizin Berlin Berlin Germany; ^4^ Division of Cardiovascular Medicine Vanderbilt University Medical Center Nashville Tennessee USA; ^5^ Center for Pulmonary Hypertension Thoraxklinik Heidelberg gGmbH at Heidelberg University Hospital and Translational Lung Research Center Heidelberg (TLRC), German Center for Lung Research (DZL) Heidelberg Germany; ^6^ Laboratory for Molecular Genetic Diagnostics, Institute of Human Genetics Heidelberg University Heidelberg Germany; ^7^ Division of Pulmonary and Critical Care Medicine Mayo Clinic Rochester Minnesota USA; ^8^ Department of Cardiovascular Medicine Mayo Clinic Rochester Minnesota USA; ^9^ Division of Pulmonary, Allergy, and Critical Care, Vera Moulton Wall Center for Pulmonary Vascular Disease Stanford University Stanford California USA; ^10^ Division of Cardiology Fondazione IRCCS Policlinico San Matteo Pavia Italy; ^11^ Department of Medicine Cardiovascular Research Center, Brown University Health Cardiovascular Institute, The Warren Alpert Medical School of Brown University Providence Rhode Island USA; ^12^ Sunshine Coast University Hospital Birtinya Queensland Australia; ^13^ 1st Department of Critical Care and Pulmonology Medicine National and Kapodistrian University of Athens School of Medicine, ‘Evangelismos’ Hospital Athens Greece; ^14^ 1st Department of Cardiology Aristotle University of Thessaloniki Thessaloniki Greece; ^15^ Department of Medicine Division of Pulmonary and Critical Care Medicine, Johns Hopkins University School of Medicine Baltimore Maryland USA; ^16^ Clinic III for Internal Medicine (Department of Cardiology) Heart Center at the University Hospital Cologne Cologne Germany; ^17^ Hospital Clinico de Barcelona Barcelona Spain; ^18^ Amsterdam UMC Amsterdam The Netherlands; ^19^ Department of Respiratory Medicine The Alfred Hospital Melbourne Victoria Australia; ^20^ Royal Papworth Hospital Cambridge Cambridge UK; ^21^ University of Health Sciences Lahore Pakistan; ^22^ University of Pittsburgh Pittsburgh Pennsylvania USA; ^23^ Hospital Universitario 12 de Octubre Madrid Spain; ^24^ University of Cordoba Córdoba Argentina; ^25^ Department of Medicine, Victor Phillip Dahdaleh Heart & Lung Research Institute University of Cambridge Cambridge UK; ^26^ Fundacion Cardiovascular de Colombia Colombia; ^27^ Division of Respiratory Medicine, Lung Research Cluster Medical University of Graz Graz Austria; ^28^ Royal Hobart Hospital Hobart Australia; ^29^ Universidad Nacional de La Plata La Plata Argentina; ^30^ University of Cincinnati Cincinnati Ohio USA; ^31^ Ludwig‐Maximillians‐University Munich Munich Germany; ^32^ Milpark Hospital Johannesburg South Africa; ^33^ Department of Internal Medicine II Division of Respiratory Medicine, University Medical Centre Hamburg‐Eppendorf Hamburg Germany; ^34^ Medical Department I Division of Pulmonology, University Hospital Carl Gustav Carus of Technical University Dresden Dresden Germany; ^35^ National Heart and Lung Institute Imperial College London London UK; ^36^ Sheffield Pulmonary Vascular Disease Unit Royal Hallamshire Hospital, University of Sheffield and National Institute for Health and Care Research Sheffield Biomedical Research Centre Sheffield UK; ^37^ Murdoch Children's Research Institute University of Melbourne Melbourne Victoria Australia; ^38^ Jagiellonian University Medical College Kraków Poland; ^39^ Department of Cardiology Pulmonary Hypertension Center, NHO Okayama Medical Center Okayama Japan; ^40^ Royal Prince Alfred Hospital Camperdown Australia; ^41^ Fiona Stanley Hospital Murdoch Australia; ^42^ Division of Pulmonology University Hospital Zurich Zürich Switzerland; ^43^ Institute of Medical Informatics RWTH Aachen University Aachen Germany; ^44^ Department of Infectious Diseases and Respiratory Medicine Charité‐Universitätsmedizin Berlin, Corporate Member of Freie Universität Berlin and Humboldt‐Universität zu Berlin Berlin Germany; ^45^ Clinica Cardiovid Medellín Colombia; ^46^ Clinica Neumologica del Pacifico Cali Colombia; ^47^ Pulmonary Vascular Research Institute (PVRI) Canterbury UK; ^48^ Cologne Cardiovascular Research Center (CCRC) Hospital Cologne and Medical Faculty, Heart Center at the University, University of Cologne Cologne Germany; ^49^ Cleveland Clinic Abu Dhabi Abu Dhabi United Arab Emirates; ^50^ Houston Methodist Hospital Houston Texas USA; ^51^ P.Shupik National University of the Healthcare of Ukraine Kyiv Ukraine; ^52^ Gold Coast University Hospital Southport Australia; ^53^ Department of Medicine University of Minnesota Minneapolis Minnesota USA; ^54^ George Emil Palade University of Medicine Târgu Mureș Romania; ^55^ Strazhensku Cardiology Institute Kiev Kiev Ukraine; ^56^ The Royal Children's Hospital Parkville Australia; ^57^ Faculty of Medicine, Nursing and Health Sciences, Central Clinical School Monash University Melbourne Victoria Australia; ^58^ Kerckhoff Klinik and German Centre for Lung Research (DZL/UGMLC) Bad Nauheim Germany; ^59^ Instituto Nacional de Cardiologia Ignacio Chavez, Ciudad de Mexico; ^60^ Department of Pulmonary and Critical Care Medicine Center of Respiratory Medicine, China‐Japan Friendship Hospital Beijing China; ^61^ National Center for Respiratory Medicine Beijing China; ^62^ State Key Laboratory of Respiratory Health and Multimorbidity Beijing China; ^63^ National Clinical Research Center for Respiratory Diseases Beijing China; ^64^ Institute of Respiratory Medicine Chinese Academy of Medical Sciences Beijing China

## Abstract

In 567 PH‐COPD patients from the PVRI GoDeep Meta‐Registry, ACE inhibitor use was associated with improved survival only in severe PH (PVR > 5 WU), supporting prospective trials targeting this high‐risk subgroup.

## 1 Introduction

Pulmonary hypertension (PH) is characterized by increased right ventricular afterload and encompasses a heterogeneous group of associated diseases [1]. Chronic lung diseases are among the most prevalent causes of PH worldwide [1]. Whereas inhaled Treprostinil has recently been FDA‐approved for PH associated with interstitial lung disease, no PH‐targeting therapies are available for PH associated with chronic obstructive lung disease (PH‐COPD) [2, 3].

Recently, Ozaltin et al. reported that angiotensin‐converting enzyme inhibitor (ACEi) therapy was associated with reduced mortality in idiopathic pulmonary fibrosis (IPF), but not in COPD patients [4]. Beyond the direct role of ACE in chronic lung disease pathobiology, both experimental and clinical studies indicate that ACE contributes to pulmonary vascular remodeling and right ventricular dysfunction [5]. Notably, a key difference between the IPF and COPD cohorts analyzed by Ozaltin et al. is the prevalence of PH: whereas approximately 5% of IPF patients (7% in the treated group) had documented PH, only 22 of 3579 COPD patients (0.6%) were diagnosed with PH in that study [4].

Given the well‐established involvement of the renin−angiotensin−aldosterone system in pulmonary vascular remodeling and endothelial dysfunction, it is plausible that the survival benefit observed in IPF was partly mediated via effects on the pulmonary vasculature and right heart function [5]. This raises the possibility that any analogous benefit in COPD may have been obscured by the very low prevalence of PH in the analyzed cohort.

Thus, we analyzed the association between ACEi therapy and survival in patients with invasively diagnosed PH‐COPD in the PVRI GoDeep meta‐registry.

## 2 Methods

### 2.1 Patient Selection

Patients invasively diagnosed with PH‐COPD from the PVRI GoDeep meta‐registry, originating from the PH centers in Giessen, Nashville, Heidelberg, Rochester, and Stanford, were retrospectively analyzed [6]. Diagnosis of PH‐COPD was established at the participating PH expert centers according to the international guideline recommendations applicable at the time of diagnosis based on the comprehensive clinical assessment performed at each center. Further inclusion criteria were: (i) age ≥ 18 years and (ii) the availability of hemodynamic parameters, including mean pulmonary artery pressure (mPAP), pulmonary vascular resistance (PVR), and systolic pulmonary arterial pressure (sPAP) assessed at the time of diagnosis. For survival analyses, survival time was defined as the time from diagnosis of PH for patients who were not treated with ACE inhibitors, and as the time from initiation of ACE inhibitor treatment for those who started treatment after PH diagnosis, to reduce immortal time bias. The PVRI GoDeep registry was approved by the University of Giessen Ethics Committee and the relevant local ethics committees (NCT05329714).

### 2.2 Statistical Analysis

Data were analyzed in R version 4.5.2 using the packages survival (version 3.8‐3), rms (version 8.1‐0), and splines (version 4.5.2). Continuous variables were summarized as medians [Q1, Q3]. For individual variables (BMI, FEV1_%predicted_, FVC _%predicted_), missing data were imputed using the mice package version 3.16.0, utilizing information from center, diagnosis decade, sex, age at diagnosis, mPAP, and PVR. The plausibility of the imputed data distribution was verified using diagnostic plots. The Cox proportional hazard models were adjusted for age (modeled using a natural spline with two degrees of freedom), BMI, FEV1%_predicted_, FVC%_predicted_, PVR, pulmonary artery wedge pressure (PAWP), PH‐targeting therapy (none, mono, dual, and triple or more), as well as strata for center, diagnosis decade, and sex. Center was additionally included as a clustering variable. In an additional analysis, the Cox proportional hazards models were further adjusted for the cardiovascular risk factors cardiac diseases, arterial hypertension, diabetes, and obesity. Sample size was determined by simulation‐based power analysis of the prespecified 5‐year Cox model using 1000 stratified resamples per candidate sample size with 80% power derived from a spline‐smoothed power curve. Sensitivity analyses for unmeasured confounders were carried out using *E*‐values, which quantify the minimum strength of association that an unmeasured confounder would need to have with both the exposure and the outcome in order to explain the observed effect estimate.

## 3 Results

We identified a subgroup of 567 patients diagnosed with PH‐COPD in the PVRI GoDeep meta‐registry and evaluated the association between ACEi and all‐cause mortality. Median age was 66 [59, 72] years, 244 (43%) were female, and 97 (17%) received ACEi. As expected, FEV1/FVC ratio (47 [34, 60] %), FEV1_predicted_ (45 [29, 62]%), mPAP (32 [26, 40] mmHg), cardiac output (4.7 [4.0, 5.8] L/min), PAWP (10 [7, 12] mmHg), PVR (4.7 [3.3, 6.7] WU), and WHO functional class (258 [66%] and 77 [20%] in WHO FC III and IV, respectively) corresponded to a COPD population with relevant PH.

**Figure 1:**
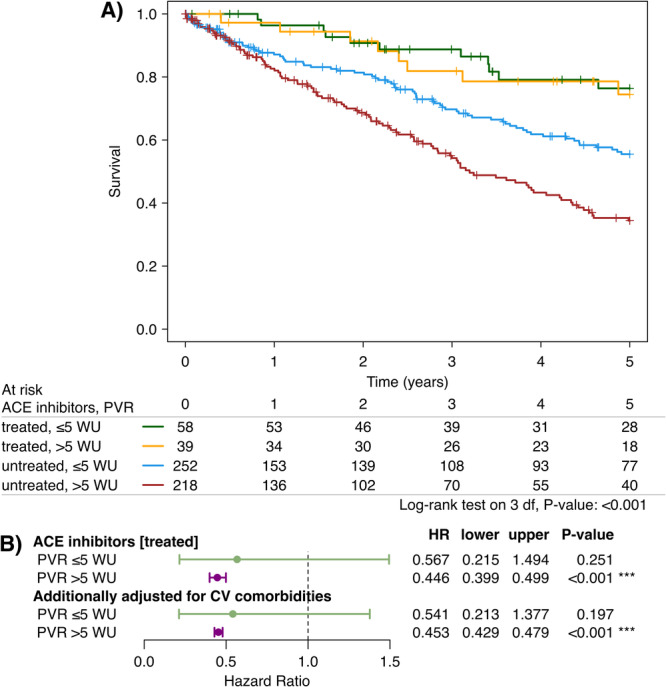
Survival and hazard ratios by ACEi and PVR. (A) Kaplan–Meier survival curves. (B) Hazard ratios comparing patients treated with ACEi versus those not treated with ACEi, for the interaction analysis between PVR (≤ 5 vs. > 5 WU) and ACEi. The Cox proportional hazard models were adjusted for BMI, FEV1_%predicted_, FVC_%predicted_, PVR, PAWP, PH‐targeting therapy (none, mono, dual, and triple or more), as well as strata for center, diagnosis decade, and sex. Center was additionally included as a clustering variable. Additionally, in a second analysis, the Cox proportional hazards models were further adjusted for cardiovascular risk factors/diseases (cardiac diseases, arterial hypertension, diabetes, and obesity). BMI = body mass index, FEV1 = forced expiratory volume in 1 second, FVC = forced vital capacity, HR = hazard ratio, lower/upper = lower and upper limits of the 95% confidence interval of the HR, PAWP = pulmonary artery wedge pressure, PVR = pulmonary vascular resistance, WU = wood units.

While in non‐severe PH‐COPD patients (i.e., PVR ≤ 5 WU) no significant association between ACEi and survival was observed, a beneficial association of ACEi and survival was noted in PH‐COPD with PVR > 5 WU (1‐, 3‐, 5‐year survival: 97%, 82%, and 75% vs. 83%, 54%, and 34%, log‐rank *p* = 0.002, Figure 1). Patients receiving ACE inhibitor therapy were more frequently PH‐targeted therapy naïve compared with those not receiving ACE inhibitors (51% vs. 64%, *χ* ^2^ *p* = 0.112). The findings remained robust in Cox‐regression analyses adjusting for FEV1_%predicted_, FVC_%predicted_, and PH‐targeting treatment, as well as after further adjustment for cardiovascular comorbidities (HR 0.453 [0.429, 0.479], *p* < 0.001, Figure 1).

*E*‐value analysis yielded an *E*‐value of 2.88, suggesting that substantial unmeasured confounding would be required to fully account for the observed association between ACE inhibitor therapy and outcome.

Based on these findings, a prospective study designed to assess the effect of ACEi on mortality in COPD patients with severe PH (PVR > 5 WU) over 5 years would require approximately 312 patients with 80% power at a two‐sided *α* of 0.05 based on the Wald *z*‐test *p* value.

## 4 Discussion

In this study, the association of ACEi therapy with survival in PH‐COPD patients enrolled in the international PVRI GoDeep meta‐registry was investigated. We observed that ACEi use was not significantly associated with improved survival in patients with non‐severe PH, whereas a significant survival benefit was seen in those with severe PH (i.e., PVR > 5 WU). This association remained robust after adjustment for lung function, PH‐targeted therapy, and cardiovascular comorbidities.

These findings suggest that the impact of ACE inhibition in COPD might be confined to patients with severe pulmonary vascular disease, highlighting PH severity as a key modifier of treatment effect. Stratifying patients by PH severity is therefore crucial when evaluating ACEi therapy in COPD populations and may partly explain observed differences in the impact of ACEi therapy between IPF and COPD patients by Ozaltin et al [4]. Given that PH‐targeted therapy has previously been associated with improved survival in PH‐COPD, the lower prevalence of such treatment in the ACE inhibitor group would be expected to bias results toward the null, thereby supporting the robustness of the observed association between ACE inhibitor therapy and improved outcomes [7]. Moreover, although residual confounding cannot be fully excluded, sensitivity analyses including adjustment for comorbidities as well as *E*‐value analysis suggested that a relatively strong unmeasured confounder would be required to fully explain the observed association.

Based on our data, a prospective study designed to assess ACEi effects on mortality in severe PH‐COPD would require approximately 312 patients for 5‐year follow‐up, assuming 80% power at a two‐sided *α* of 0.05. These estimates can inform future trial design, emphasizing the need to focus on the high‐risk severe PH‐COPD subgroup.

Mechanistically, our results align with experimental and clinical evidence that the renin–angiotensin−aldosterone system contributes to pulmonary vascular remodeling, endothelial dysfunction, and right ventricular maladaptation [8, 9]. Taken together, our findings provide a strong rationale for prospective trials evaluating ACE inhibition as a targeted intervention in severe PH‐COPD patients.

## Author Contributions

A.Y., M.F., K.W., J.S.A., E.B., C.A.E., E.G., H.R.C., R.F., A.J.S., R.T.Z., F.G, J.W., H.‐A.G., K.T., and W.S. collected the epidemiological and clinical data and contributed to the interpretation. A.Y., M.F., K.W., and J.W. processed the statistical data. A.Y. and W.S. drafted the manuscript. All authors revised the final manuscript.

## Ethics Statement

The University of Giessen/University Hospital Ethics Committee and the responsible local ethics committees have approved the PVRI‐GoDeep central data repository, listed under ClinTrial.gov (NCT05329714).

## Conflicts of Interest

A.Y. reports non‐financial support from the University of Giessen during the conduct of the study, research grants from the German Research Foundation, support for attending a meeting from OrphaCare and AOP, and personal fees from Phev, MSD, and Ferrer outside the submitted work. E.B. has received investigator‐initiated grant funding and advisory board‐related personal fees from United Therapeutics. C.A.E. received honoraria for lectures and presentations from OMT, MSD, and Ferrer, consulting fees from MSD. E.G. received fees for lectures and/or consultations from Actelion, Bayer, GSK, Janssen, MSD, Pfizer and United Therapeutics; reports grants from Acceleron, Actelion, Aerovate, Bayer, Ferrer, Gossamer, Insmed, Janssen, Keros, Liquidia, Merck, MSD, Novartis, OMT, United Therapeutics, consulting fees from Actelion, Ferrer, Janssen, Merck, MSD, honoraria from Actelion, AOP, Bayer, Ferrer, GEBRO, GSK, GWT, Janssen, MSD, OMT, Phev, and participation on advisory boards from Actelion, Ferrer, and MSD. R.F. received consulting fees from Merck Inc, Insmed, Inhibikase, and Gossamer Bio. He holds equity interests in Merck. A.J.S. received K23 grant support (K23HL151892) from NIH/NHLBI. R.Z. has a patent FK506 for treatment of PAH issued to Stanford University. H.‐A.G. has received fees from Actelion, AstraZeneca, Bayer, GSK, Janssen‐Cilag, Lilly, Novartis, OMT, Pfizer, and United Therapeutics. K.T. has received personal fees from Bayer, AstraZeneca, and Gossamer. W.S. has received consultancy fees from United Therapeutics, Tiakis Biotech AG, Liquidia, Pieris Pharmaceuticals, Abivax, Pfitzer, and Medspray BV. The other authors declare no conflicts of interest.

## Data Availability

The data that support the findings of this study are available on request from the corresponding author. The data are not publicly available due to privacy or ethical restrictions.
